# Mowing Enhances Insect Resistance in *Glycyrrhiza uralensis* by Reprogramming Volatile Profiles and Inducing Flavonoid Accumulation

**DOI:** 10.3390/insects17020211

**Published:** 2026-02-17

**Authors:** Zhenghui Guan, Wenjia Gao, Hui Duan, Xiushuang Wang

**Affiliations:** 1 College of Life Science, Shihezi University, Shihezi 832003, China; guanzhenghui2025@163.com (Z.G.); gaowenjia313@163.com (W.G.); duanhui10042000@163.com (H.D.); 2 Xinjiang Production and Construction Corps Key Laboratory of Oasis Town and Mountain-Basin System Ecology, Shihezi University, Shihezi 832003, China; 3 Ministry of Education Key Laboratory of Xinjiang Phytomedicine Resource Utilization, Shihezi University, Shihezi 832003, China

**Keywords:** *Bemisia tabaci*, *Glycyrrhiza uralensis*, volatiles, multi-omics analysis, defense mechanisms, flavonoid biosynthesis

## Abstract

Mowing is a widely used agricultural management practice, yet its role in shaping plant–insect interactions remains largely unexplored. In this study, we investigated how mowing influences resistance of licorice (*Glycyrrhiza uralensis*) to the whitefly *Bemisia tabaci* by integrating behavioral assays with volatile analysis, transcriptomics, and metabolomics. Feeding preference assays showed that adult whiteflies strongly preferred new plants over mowed plants. Developmental assays further revealed that whiteflies exhibited a prolonged egg stage and extended egg-to-adult developmental duration on mowed plants, while adult longevity was not significantly affected. Gas chromatography–mass spectrometry analysis identified 31 volatile compounds in licorice, with alcohols dominating the volatile profile of new plants and terpenoids dominating that of mowed plants. Whitefly infestation significantly increased ester compounds in both plant types. Differential volatile analysis highlighted cis-3-hexen-1-ol and trans-3-hexen-1-ol as dominant compounds in new plants, whereas 3-carene and β-pinene were predominant in mowed plants. Transcriptomic analysis revealed that mowing primarily affected genes associated with primary metabolism and ribosome-related pathways, whereas whitefly infestation induced extensive transcriptional reprogramming, including activation of flavonoid biosynthesis, flavone and flavonol biosynthesis, MAPK signaling, and plant circadian rhythm pathways. Metabolomic profiling identified substantial accumulation of flavonoids, flavonols, and isoflavonoids following whitefly feeding. Integrated multi-omics analysis identified flavonol biosynthesis as a core pathway underlying licorice defense against *B. tabaci*. Overall, this study demonstrates that mowing primes *G. uralensis* for enhanced resistance to whitefly infestation by reshaping volatile emissions, activating secondary metabolite biosynthesis, and inducing coordinated defense signaling networks. These findings provide new insights into plant–insect interactions and highlight mowing as a potential component of sustainable pest management strategies.

## Simple Summary

Whiteflies (*Bemisia tabaci*) are serious pests that damage many crops, including licorice (*Glycyrrhiza uralensis*). Finding environmentally friendly ways to reduce whitefly infestation is therefore important for sustainable agriculture. In this study, we examined whether mowing, a simple cultural practice, could enhance licorice resistance to whiteflies. We found that whiteflies strongly preferred unmowed plants, while mowed plants were less attractive. Mowing changed the chemical signals released by plants, reducing compounds that attract whiteflies and increasing terpenoids that repel them. In addition, mowed plants accumulated higher levels of defensive flavonoids after whitefly feeding. As a result, whiteflies developed more slowly on mowed plants. These findings show that mowing can naturally strengthen plant defenses against insect pests. Our study highlights mowing as a low-cost and eco-friendly strategy that could help reduce pesticide use in licorice cultivation and other cropping systems.

## 1. Introduction

The genus *Glycyrrhiza* represents one of the most widely utilized bulk medicinal herbs, with a documented history of medicinal use spanning over 2200 years [1]. Species within this genus are rich in glycyrrhizic acid, glycyrrhetinic acid, and flavonoids, which exhibit diverse pharmacological activities, including antiviral, antibacterial, neuroprotective, anti-inflammatory, antitussive, detoxifying, anti–breast cancer, anti–atopic dermatitis, anti–muscle fibrosis, and anti–prostatic hyperplasia effects [2]. Owing to these pharmacological properties, *Glycyrrhiza* has long been regarded as a cornerstone of traditional Chinese medicine. Among the species within this genus, *Glycyrrhiza uralensis* is one of the most important medicinal plants. It exhibits remarkable physiological adaptations, including tolerance to saline–alkaline soils and drought conditions, and is therefore widely used as a windbreak and sand-fixation plant in arid and semi-arid regions, highlighting its substantial ecological value [3]. However, excessive harvesting has resulted in a sharp decline in wild populations of *G. uralensis*, leading to its classification as a second-level protected plant under China’s national conservation guidelines (National Forestry and Grassland Administration, Ministry of Agriculture and Rural Affairs, 2021). Artificial cultivation has consequently become the primary strategy for meeting market demand. Nevertheless, large-scale monoculture cultivation has intensified pest and disease pressures, which significantly reduce root yield and the accumulation of bioactive compounds in *G. uralensis* [4]. Therefore, the development of sustainable cultivation systems and effective pest management strategies is essential for the long-term viability of the *Glycyrrhiza* industry.

To date, research on stress response mechanisms in *Glycyrrhiza* has predominantly focused on abiotic factors. Transcriptomic and metabolomic studies have demonstrated that drought stress in *G. uralensis* induces extensive reprogramming of genes and metabolites associated with osmotic adjustment, antioxidant defense, phenylpropanoid metabolism, and flavonoid biosynthesis [5]. Similarly, salt stress markedly upregulates genes involved in phytosterol, oleanane, and lupane saponin biosynthesis, as well as flavonoid and triterpenoid metabolic pathways [6,7]. In contrast, studies addressing biotic stress responses, particularly those associated with insect herbivory, remain limited. This imbalance highlights a critical gap in understanding how *Glycyrrhiza* responds to insect-mediated stress under cultivation conditions.

The silverleaf whitefly, *Bemisia tabaci*, is a globally distributed phloem-feeding insect and is considered one of the most destructive agricultural pests worldwide [8]. This highly polyphagous species infests more than 900 plant species, including numerous economically important crops and ornamental plants cultivated in both field and greenhouse systems [9,10,11]. Moreover, *B. tabaci* is capable of transmitting over 300 plant pathogenic viruses, earning its designation as a “super pest” in agricultural ecosystems. Severe infestations of *B. tabaci* have been reported in *Glycyrrhiza* species, particularly *G. uralensis*, where damage levels can reach category 4, corresponding to infestation densities exceeding 50 individuals per 100 cm^2^ of leaf area [12]. Such infestations cause direct damage through phloem feeding and indirectly exacerbate plant injury by facilitating virus transmission and promoting sooty mold development, posing serious ecological and economic threats to *G. uralensis* cultivation.

Mowing is a common agronomic practice that imposes recurrent mechanical disturbance on plants and acts as a selective pressure influencing growth, metabolism, and stress acclimation [13,14]. Mechanical injury caused by mowing rapidly induces the emission of volatile organic compounds (VOCs), including aldehydes, terpenoids, and sesquiterpenes, which play important roles in shaping insect host-selection behavior [15]. However, mowing-induced mechanical damage fundamentally differs from damage caused by insect feeding. Importantly, mowing does not introduce insect-derived elicitors, and the defense responses it activates primarily reflect wound-mediated signaling and defense priming rather than herbivore-specific recognition.

Plants are constantly exposed to herbivorous insects and have evolved diverse defense strategies to mitigate insect damage. These strategies include constitutive and inducible defenses, such as physical barriers, secondary metabolites, and complex signaling networks that regulate gene expression and metabolite accumulation in response to herbivory [16]. In phloem-feeding insects such as *B. tabaci*, plant defense responses are elicited not only by physical injury caused by stylet penetration but also by salivary elicitors delivered during feeding. These elicitors are recognized by plant defense systems at the molecular level and activate signaling pathways that differ markedly from those induced by mechanical damage alone. Consequently, insect feeding often induces distinct biosynthetic responses, leading to pronounced changes in chemical profiles, transcriptomic patterns, and phytohormone signaling networks. Plant defense against insect herbivory is therefore a dynamic and multidimensional process, initiated by early signaling events such as membrane depolarization, calcium influx, reactive oxygen species (ROS) generation, and protein phosphorylation, followed by phytohormone-mediated signal integration and transcriptional reprogramming that culminates in the production of defensive secondary metabolites [17].

In plant–insect interactions, effective defense relies on the coordinated regulation of early immune signaling and downstream metabolic responses. Genes involved in phytohormone signaling, terpenoid biosynthesis, and flavonoid metabolism play pivotal roles in shaping plant resistance to herbivorous insects. Salicylic acid–mediated signaling is particularly important in defense against phloem-feeding insects, with *pathogenesis-related protein 1* (*PR1*) widely recognized as a conserved molecular marker of systemic acquired resistance [18]. In parallel, flavonoid- and isoflavonoid-based defenses represent a major class of specialized metabolites contributing to insect resistance, especially in leguminous plants. Chalcone synthase (*CHS*), which catalyzes the first committed step of the flavonoid biosynthetic pathway, is essential for stress-induced accumulation of flavonoids and phytoalexins [19]. Moreover, recent studies have identified 2-hydroxyisoflavanone 4′-O-methyltransferase (*HI4′OMT*) as a key regulatory enzyme controlling the biosynthesis of formononetin-derived isoflavonoids, which constitute important legume-specific chemical defenses [20]. In addition to flavonoid-related pathways, terpenoids synthesized via the methylerythritol phosphate (MEP) pathway frequently function as repellents or indirect defense signals [21], while jasmonic acid and salicylic acid signaling pathways integrate wound-induced and elicitor-mediated responses [22]. Together, these pathways and regulatory genes form an interconnected defense network linking signal perception with specialized metabolite biosynthesis. Accordingly, these defense-related pathways and representative regulatory genes were selected as focal points for transcriptomic, metabolomic, and gene expression analyses in the present study.

In this study, the *B. tabaci*–*G. uralensis* interaction was employed as a model system to investigate the regulatory mechanisms underlying mowing-induced insect resistance in *G. uralensis* using multi-omics approaches. By integrating mowing treatment with whitefly infestation, this work aims to disentangle defense responses driven by mechanical damage from those mediated by insect-derived elicitors. The results provide new insights into the biotic stress responses of *Glycyrrhiza* and offer a theoretical foundation for the development of insect-resistant cultivars and ecologically sustainable pest management strategies.

## 2. Materials and Methods

### 2.1. Plant Cultivation and Growth Conditions

Seeds of *Glycyrrhiza uralensis* were obtained from the College of Life Sciences, Shihezi University, China. Plants were cultivated in plastic pots (15 cm height × 15 cm diameter) filled with a seedling substrate mixture consisting of commercial nutrient substrate, vermiculite, and perlite at a volume ratio of 3:1:1.

The commercial nutrient substrate (Pindstrup, Ryomgaard, Denmark) was composed primarily of coconut coir (0–5 mm), peat moss (0–10 mm), vermiculite (1–3 mm), and perlite, with proportions of 35%, 30%, 15%, and 20% (*v*/*v*), respectively. A wetting agent (100 mL·m^−3^, diluted 1:2500) and a starter fertilizer (1.5 kg·m^−3^) were incorporated by the manufacturer. The substrate pH was adjusted to 5.5–6.5, and electrical conductivity was maintained below 1.0 mS·cm^−1^ to ensure suitable nutrient availability for licorice growth.

### 2.2. Mowing Treatment and Experimental Material Preparation

Mowing treatment was applied when new (non-mowed) plants reached approximately 20 cm in height. Aboveground tissues were cut using sterilized scissors, leaving a residual stem height of 2 cm above the soil surface. Mowed plants were returned to the incubator and maintained under the same environmental conditions.

Simultaneously, *G. uralensis* seeds were newly sown under identical cultivation conditions to generate a cohort of primary plants. This design ensured that regrowing mowed plants and newly established plants experienced comparable growth durations and environmental conditions. After approximately two months, both plant groups reached similar developmental stages and were defined as mowed plants (M) and new plants (N) for subsequent experiments.

### 2.3. Insect Rearing

The test insect *Bemisia tabaci* was obtained from the Tobacco Cultivation Laboratory, College of Life Sciences, Shihezi University, China. The colony originated from field populations collected in Shihezi City, Xinjiang, and was reared on wild-type tobacco plants under laboratory conditions for more than five generations. Rearing conditions were maintained at 26 ± 1 °C, 60 ± 5% relative humidity, and a 16 h light/8 h dark photoperiod.

Although molecular identification of cryptic species was not conducted in this study, previous regional surveys have shown that field populations from Shihezi City consist exclusively of the MEAM1 cryptic species, whereas MED predominates in other areas of Xinjiang. Based on these published data, the experimental population used here was most likely MEAM1 [23].

### 2.4. Feeding Preference Assay

Feeding preference assays were performed in nylon mesh insect-rearing cages (60 × 60 × 60 cm). One pot of new growth and one pot of mowed *G. uralensis* with similar growth status were randomly placed at equal distances within each cage. A total of 100 adult *B. tabaci* (♀:♂ ≈ 1:1), starved for 4 h prior to the experiment, were released at the midpoint between the two plants and allowed to freely select host plants.

The number of adults settled on each plant was recorded at 3, 6, 9, 12, 24, 36, 48, 60, and 72 h after release. Each cage represented one independent experimental unit. The entire experiment was independently repeated three times using separate cages and newly prepared plants, and each experimental run was considered one biological replicate (*n* = 3).

At each observation time, feeding preference was analyzed using a chi-square (χ^2^) goodness-of-fit test to determine whether the distribution of whiteflies deviated from a 1:1 random expectation between new and mowed plants.

### 2.5. Measurement of Developmental Duration and Longevity

To evaluate the effects of mowing on whitefly development, one healthy new plant and one mowed plant were selected for each replicate. On a leaf at the same node position, 20 newly emerged adults (2–3 days old; ♀:♂ ≈ 1:1) were introduced and confined using a nonwoven fabric bag. After 24 h, all adults were removed, leaving only eggs on the leaf surface.

Developmental progress was monitored daily, and the duration of each life stage (egg, nymphal instars, and adult emergence) was recorded. Newly emerged adults were transferred to fresh plants of the corresponding treatment, and adult longevity was recorded until death. Each treatment was independently replicated three times. Differences in developmental duration and longevity between treatments were analyzed using Student’s *t*-test (*p* < 0.05).

### 2.6. Volatile Organic Compound (VOC) Analysis

#### 2.6.1. Experimental Design and Plant Treatments

VOC analysis was conducted using a factorial design with two factors: mowing treatment (new vs. mowed plants) and insect infestation (with vs. without *B. tabaci*). For each experimental run, two pots of new plants and two pots of mowed plants were individually placed into insect-rearing cages (40 × 40 × 40 cm), with one plant per cage.

In two cages, 50 adult *B. tabaci* (♀:♂ ≈ 1:1), starved for 4 h, were released and allowed to feed for 24 h. The remaining two cages served as non-infested controls. Four treatments were established: N, M, N-Bt, and M-Bt. After 24 h, fresh leaves were collected. Leaf material from plants within the same treatment and experimental run was pooled to form one sample.

The entire experiment was independently repeated three times using newly prepared plants and cages, resulting in three biological replicates per treatment (*n* = 3; total samples = 12). For each sample, 0.50 g of fresh leaves was finely cut and immediately transferred into a 20 mL headspace vial.

#### 2.6.2. HS-GC-MS Analysis of VOCs

VOCs were analyzed using headspace gas chromatography–mass spectrometry (HS-GC-MS) on an Agilent 19091S-433UI system (Agilent Technologies, Santa Clara, CA, USA). Headspace conditions were set to 95 °C for 25 min with an injection volume of 1 mL. Separation was performed on an HP-5MS capillary column using helium as the carrier gas (1 mL/min). The oven temperature was programmed from 60 °C (2 min) to 200 °C at 5 °C/min.

Mass spectra were acquired under electron ionization (70 eV) over an *m/z* range of 30–500. Compounds were identified using the NIST14 and Flavor databases. Relative contents were calculated by peak area normalization. PCA and OPLS-DA were performed using SIMCA 18.0. Compounds with *p* < 0.05 (ANOVA) and VIP ≥ 1 were considered significantly different.

### 2.7. Transcriptome and Metabolomic Analyses

#### 2.7.1. Transcriptome Sequencing and Analysis

Leaves from the four treatments were collected after 24 h of infestation, frozen in liquid nitrogen, and stored at −80 °C. Total RNA was extracted using the MJZol kit (Magen Biotech, Guangzhou, China) and assessed for quality. Twelve cDNA libraries (three biological replicates per treatment) were constructed and sequenced on an Illumina HiSeq 4000 platform (Illumina Inc., San Diego, CA, USA).

Clean reads were aligned to the *G. uralensis* reference genome using HISAT2. Differentially expressed genes were identified using DESeq2 with thresholds of |log_2_ fold change| > 2 and adjusted *p* < 0.05. GO and KEGG enrichment analyses were conducted.

#### 2.7.2. Metabolomic Analysis

Metabolomic profiling of *G. uralensis* leaves following 24 h of B. tabaci infestation was conducted using liquid chromatography-tandem mass spectrometry (LC-MS/MS). Chromatographic separation was achieved on a BEH C18 column (100 mm × 2.1 mm, 1.8 µm) with an injection volume of 10 μL. The mobile phase consisted of 0.1% formic acid in water (A) and 0.1% formic acid in a 1:1 acetonitrile-isopropanol mixture (B). The gradient elution was programmed as follows: 95% A to 80% A (5% B to 20% B) from 0 to 3 min; 80% A to 5% A (20% B to 95% B) from 3 to 9 min; isocratic 5% A (95% B) from 9 to 13 min; return to 95% A (5% B) from 13.0 to 13.1 min; and re-equilibration at 95% A from 13.1 to 16 min. The flow rate was 0.40 mL/min and the column temperature was maintained at 40 °C.

Mass spectrometry was performed in both positive and negative ion modes across *m/z* 50–1000. The ion spray voltage was set to 5000 V (positive) and 4000 V (negative), with a declustering potential of 80 V. Nebulizer gas (GS1) and auxiliary gas (GS2) pressures were 50 psi, curtain gas pressure was 30 psi, and the ion source temperature was 500 °C. Collision energy was optimized between 20 and 60 V.

Multivariate analysis (PCA and OPLS-DA) was carried out using the ropls package (v1.6.2) in R. Differentially accumulated metabolites were identified based on a variable importance in projection (VIP) > 1 from OPLS-DA and *p* < 0.05 from Student’s *t*-test. Enrichment analysis of KEGG pathways was subsequently performed on the selected metabolites.

### 2.8. qRT-PCR Analysis

To validate transcriptomic results, quantitative real-time PCR (qRT-PCR) was performed using total RNA extracted from the four experimental treatments (N, M, N-Bt, and M-Bt). First-strand cDNA synthesis was conducted using the HiScript II First Strand cDNA Synthesis Kit (Vazyme, Nanjing, China). qRT-PCR reactions were performed using the Real Master Mix (SYBR Green) Kit (Vazyme, Nanjing, China) on a LightCycler 96 Real-Time PCR System (Roche, Basel, Switzerland).

Three target genes were selected for validation (Table S1), and the JAR1 gene was used as the internal reference for normalization. Each reaction was carried out in a 20 μL volume and analyzed in triplicate. Relative gene expression levels were calculated using the 2^−ΔΔCt^ method.

### 2.9. Statistical Analysis

All statistical analyses were performed using IBM SPSS Statistics 23.0 (IBM Corp., Armonk, NY, USA), unless otherwise stated. For the feeding preference assay, chi-square (χ^2^) goodness-of-fit tests were applied at each observation time to determine whether the distribution of adult B. tabaci between new and mowed plants deviated from a 1:1 random expectation. Differences in developmental duration and adult longevity between treatments were analyzed using Student’s *t*-test. For volatile organic compound (VOC) data, relative compound abundances were analyzed using one-way analysis of variance (ANOVA), and compounds with *p* < 0.05 were considered significantly different. Multivariate analyses, including principal component analysis (PCA) and orthogonal partial least squares discriminant analysis (OPLS-DA), were conducted using SIMCA 18.0. Compounds with variable importance in projection (VIP) ≥ 1 and *p* < 0.05 were defined as differential VOCs. For transcriptomic data, differential gene expression analysis was performed using DESeq2, with |log_2_ fold change| > 2 and adjusted *p* < 0.05 as thresholds. For metabolomic data, PCA and OPLS-DA were conducted using the ropls package (v1.6.2) in R. Differentially accumulated metabolites were identified based on VIP > 1 and *p* < 0.05 from Student’s *t*-test.

All graphical representations were generated using Origin 2018 (OriginLab Corp., Northampton, MA, USA). Differences were considered statistically significant at *p* < 0.05 unless otherwise specified.

## 3. Results

### 3.1. Feeding Preference of B. tabaci for New and Mowed G. uralensis

**Figure 1:**
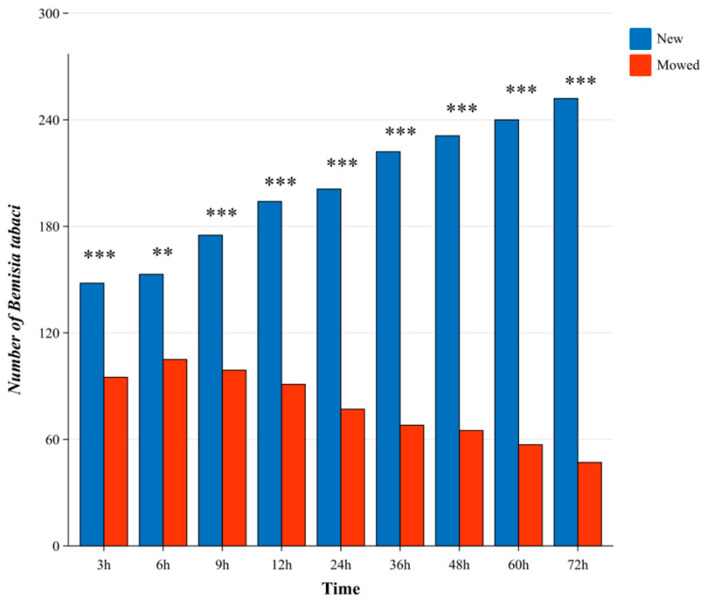
Abundance of *B. tabaci* adults on new versus mowed *G. uralensis* at different time points. Blue bars represent the number of *B. tabaci* adults on new *G. uralensis* plants, and orange bars represent those on mowed *G. uralensis* plants. Asterisks indicate significant differences between the two plant treatments at the corresponding time points: ** *p* < 0.01, *** *p* < 0.001.

The host selection behavior of *Bemisia tabaci* adults differed significantly between new and mowed *G. uralensis* plants (Figure 1). During the first 6 h after release, the number of adults settled on both plant types increased. After 6 h, significantly more *B. tabaci* adults were observed on new plants than on mowed plants (*p* < 0.01). Over time, the number of adults on new plants continued to increase, whereas the number on mowed plants gradually declined. This resulted in a progressively greater divergence in adult distribution between the two plant types throughout the observation period (*p* < 0.001).

### 3.2. Developmental Duration and Longevity of B. tabaci

The developmental duration of *B. tabaci* differed between new and mowed *G. uralensis* plants (Table 1). The egg stage was longer on mowed plants than on new plants, leading to an extended egg-to-adult developmental period on mowed plants (23.23 ± 0.25 d) compared with new plants (22.12 ± 0.13 d). In contrast, the durations of individual nymphal instars showed only minor differences between treatments. Adult longevity did not differ markedly between new and mowed plants, indicating that mowing primarily affected pre-adult development.

### 3.3. Volatile Organic Compounds Induced by B. tabaci Feeding

GC–MS analysis identified a total of 31 volatile organic compounds in *G. uralensis*, including 4 alcohols, 8 aldehydes, 5 esters, 2 acids, 11 terpenes, and 1 other compound (Table 2). In unstressed plants, alcohols accounted for the largest proportion of total volatiles in new plants (40.7%), whereas terpenes dominated the volatile profile of mowed plants (57.1%). Following *B. tabaci* infestation, ester content increased markedly in both new and mowed plants, reaching 39.8% in N-Bt and 53.4% in M-Bt, respectively.

Based on the criteria of *p* < 0.05 and VIP > 1, six differential volatile compounds were identified: cis-3-hexen-1-ol, trans-3-hexen-1-ol, (Z)-3-hexenyl acetate, 3-carene, β-pinene, and cis-3-hexenyl 2-methylbutanoate. In new plants, cis-3-hexen-1-ol and trans-3-hexen-1-ol were the most abundant compounds, accounting for 20.0% and 18.1% of total volatiles, respectively. In mowed plants, 3-carene and β-pinene were the dominant components, accounting for 18.7% and 21.4%, respectively. After *B. tabaci* infestation, the relative contents of (Z)-3-hexenyl acetate and cis-3-hexenyl 2-methylbutanoate increased significantly in both N-Bt and M-Bt groups.

### 3.4. Transcriptome Analysis under Different Treatments of B. tabaci

**Figure 2:**
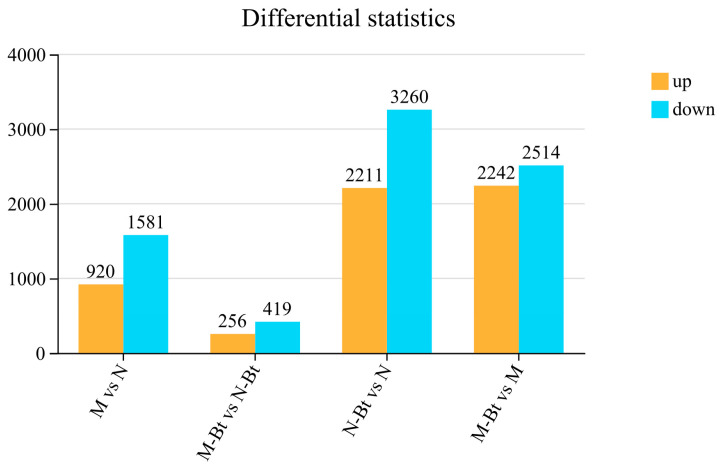
Number of differentially expressed genes (DEGs) in pairwise comparisons between different *G. uralensis* sample groups. Orange bars denote the number of significantly upregulated DEGs, while blue bars denote the number of significantly downregulated DEGs. The compared groups (M vs. N, M-Bt vs. N-Bt, N-Bt vs. N, M-Bt vs. M) correspond to the following sample treatments: M = mowed *G. uralensis* plants; N = new *G. uralensis* plants; Bt = plants infested by *B. tabaci*. DEGs were identified with the thresholds of |log_2_ fold change| > 2 and *p* < 0.05.

In this study, transcriptome sequencing of 12 samples was performed, generating a total of 90.72 Gb of clean data. Each sample yielded more than 6.08 Gb of clean data, with Q30 base percentages exceeding 95.56%. The alignment rates of clean reads to the designated *Glycyrrhiza* reference genome ranged from 92.58% to 93.86%, indicating that both the sequencing data and the chosen reference genome are of high quality and suitable for analysis (Table S2). Principal component analysis (PCA) was performed on all samples to assess their dispersion (Figure S1). The results showed that the first principal component (PC1) and the second principal component (PC2) accounted for 34.86% and 18.72% of the variance, respectively. The samples exhibited significant dispersion, indicating high reliability of the analytical results. Using the criteria of |log_2_ fold change| > 2 and *p* < 0.05 to identify DEGs, the analysis revealed the following: 2501 DEGs were identified in the M vs. N group, including 920 significantly upregulated genes and 1581 significantly downregulated genes; 675 DEGs were identified in the M-Bt vs. N-Bt group, including 256 significantly upregulated genes and 419 significantly downregulated genes. 4756 DEGs were identified in the M-Bt vs. M group, including 2242 significantly upregulated genes and 2514 significantly downregulated genes. 5471 DEGs were identified in the N-Bt vs. N group, including 2211 significantly upregulated genes and 3260 significantly downregulated genes. Notably, the number of DEGs between new and mowed plants under the same *B. tabaci* treatment was significantly lower than that of the same plant type under different *B. tabaci* treatments. Therefore, genetic mechanisms may be more responsive to *B. tabaci* infestation than to mowing treatment (Figure 2).

**Figure 3:**
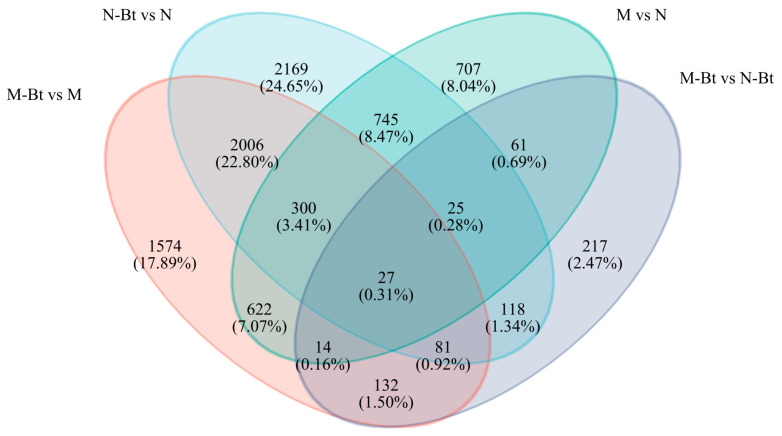
Venn diagram of differentially expressed genes (DEGs) among four pairwise comparison groups of *G. uralensis* samples. The groups in the diagram correspond to the following pairwise comparisons: M vs. N (mowed vs. new *G. uralensis* plants), M-Bt vs. N-Bt (mowed *G. uralensis* infested by *B. tabaci* vs. new *G. uralensis* infested by *B. tabaci*), M-Bt vs. M (mowed *G. uralensis* infested by *B. tabaci* vs. non-infested mowed *G. uralensis*), and N-Bt vs. N (new *G. uralensis* infested by *B. tabaci* vs. non-infested new *G. uralensis*).

Venn diagram analysis (Figure 3) revealed significant unique and common DEGs among the different treatment groups. Specifically, 707 unique DEGs were identified in the M vs. N group, 217 unique DEGs in the M-Bt vs. N-Bt group, 1574 unique DEGs in the M-Bt vs. M group, and 2169 unique DEGs in the N-Bt vs. N group. Additionally, 27 common DEGs were found between all groups. These identified DEGs may be involved in the response mechanisms of *G. uralensis* to *B. tabaci* feeding stress.

### 3.5. Functional Enrichment Analysis of the Identified DEGs

**Figure 4:**
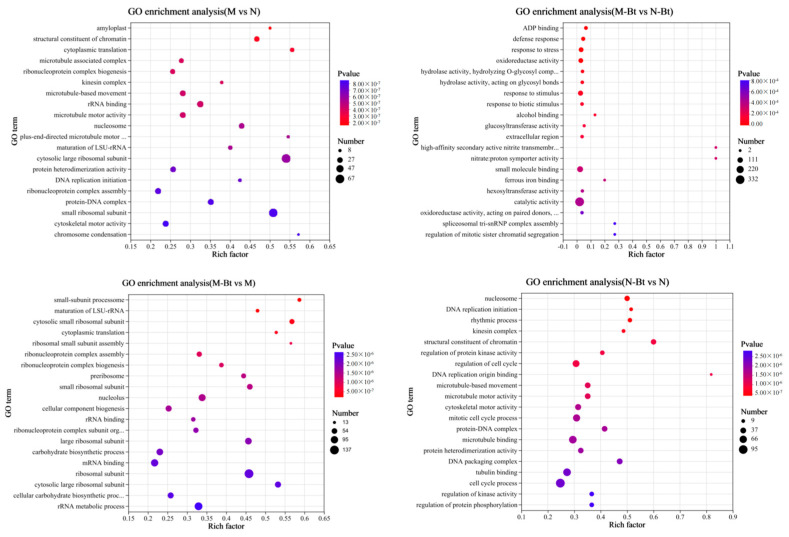
GO enrichment analysis bubble plots of differentially expressed genes (DEGs) in four pairwise comparison groups of *G. uralensis* samples. The four subplots (arranged top-left to bottom-right) correspond to the following pairwise comparisons: M vs. N, M-Bt vs. N-Bt, M-Bt vs. M, and N-Bt vs. N. In each bubble plot: The *x*-axis represents the Rich factor (ratio of DEGs annotated to a GO term to the total background genes annotated to that term, reflecting enrichment degree); The *y*-axis lists significantly enriched GO terms (screened with *p* < 0.05); Bubble color corresponds to *p*-value (darker red indicates a smaller *p*-value, i.e., more significant enrichment); Bubble size represents the number of DEGs annotated to the corresponding GO term.

Using a statistical significance threshold of *p* < 0.05, significant GO terms and KEGG pathways were identified. The identified DEGs of the M vs. N, M-Bt vs. N-Bt, M-Bt vs. M, and N-Bt vs. N groups were significantly enriched in 300, 175, 282, and 262 GO terms related to biological processes, respectively. The biological processes involving DEGs differed significantly among the treatment groups (Figure 4). Specifically, in the M vs. N group, DEGs were mainly enriched in processes such as cytoplasmic translation, ribonucleoprotein complex biogenesis, microtubule-based movement, maturation of LSU-rRNA, and DNA replication initiation. In the M-Bt vs. N-Bt group, DEGs were primarily enriched in defense response, response to stress, response to stimulus, and response to biotic stimulus. In the M-Bt vs. M group, DEGs were mainly enriched in maturation of LSU-rRNA, cytoplasmic translation, ribosomal small subunit assembly, and ribonucleoprotein complex biogenesis. In the N-Bt vs. N group, DEGs were mainly enriched in DNA replication initiation, rhythmic process, regulation of protein kinase activity, and regulation of cell cycle. Additionally, DEGs from all four groups were significantly enriched in the following GO terms: glucan metabolic process, cellular carbohydrate metabolic process, cellular polysaccharide metabolic process, and glucosyltransferase activity.

**Figure 5:**
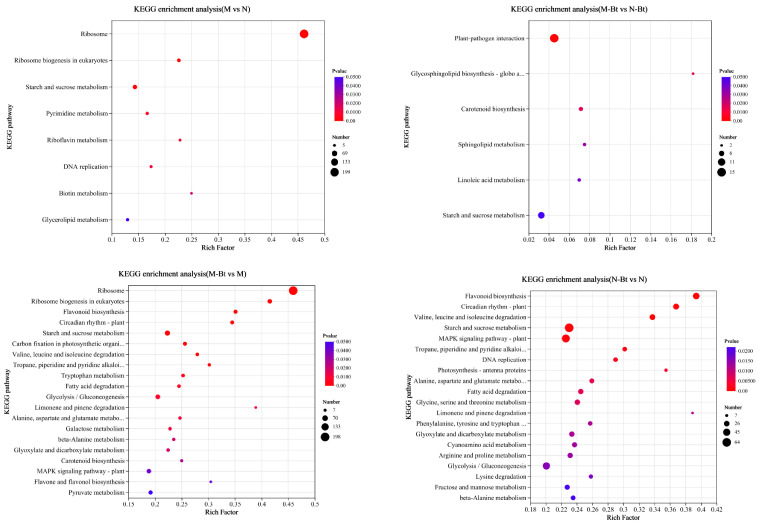
KEGG pathway enrichment analysis bubble plots of differentially expressed genes (DEGs) in four pairwise comparison groups of *G. uralensis* samples. The four subplots (arranged top-left to bottom-right) correspond to the following pairwise comparisons: M vs. N, M-Bt vs. N-Bt, M-Bt vs. M, and N-Bt vs. N. In each bubble plot: The *x*-axis represents the Rich factor (ratio of DEGs annotated to a KEGG pathway to the total background genes annotated to that pathway, reflecting enrichment degree); The *y*-axis lists significantly enriched KEGG pathways (screened with *p* < 0.05); Bubble color corresponds to *p*-value (darker red indicates a smaller *p*-value, i.e., more significant enrichment); Bubble size represents the number of DEGs annotated to the corresponding KEGG pathway.

KEGG pathway analysis was performed to map the DEGs to relevant metabolic pathways, revealing their functional roles in biological processes. The identified DEGs of the M vs. N, M-Bt vs. N-Bt, M-Bt vs. M, and N-Bt vs. N were significantly enriched in 9, 6, 20, and 26 KEGG pathways, respectively (Figure 5). Under the same treatment conditions, the metabolic pathways involving DEGs differed significantly between new and mowed *G. uralensis* plants. Specifically, in the M vs. N group, DEGs were mainly enriched in pathways such as ribosome, ribosome biogenesis in eukaryotes, starch and sucrose metabolism, pyrimidine metabolism, and riboflavin metabolism. In the M-Bt vs. N-Bt group, DEGs were primarily enriched in plant-pathogen interaction, carotenoid biosynthesis, globo and isoglobo series glycosphingolipid biosynthesis, linoleic acid metabolism, and sphingolipid metabolism. The metabolic pathways enriched by DEGs in M-Bt vs. M were significantly similar to those in N-Bt vs. N. Both groups showed significant enrichment in pathways such as flavonoid biosynthesis, plant circadian rhythm regulation, starch and sucrose metabolism, flavone and flavonol biosynthesis, and mitogen-activated protein kinases (MAPK) signaling pathway. Specifically, in the M-Bt vs. M group, DEGs were mainly enriched in ribosome, ribosome biogenesis in eukaryotes, flavonoid biosynthesis, plant circadian rhythm regulation, and starch and sucrose metabolism. In the N-Bt vs. N group, DEGs were primarily enriched in flavonoid biosynthesis, plant circadian rhythm regulation, valine, leucine, and isoleucine degradation, starch and sucrose metabolism, and MAPK signaling pathway.

### 3.6. Annotation of Metabolites

**Figure 6:**
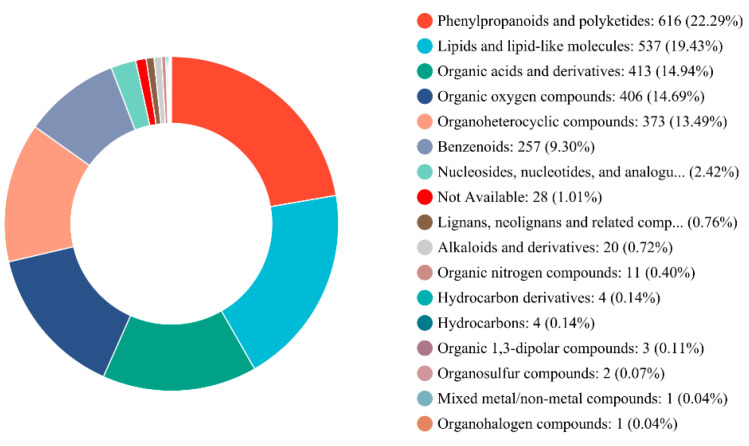
Metabolite profiling of new and mowed *G. uralensis* plants with or without *B. tabaci* infestation. Analysis was performed using LC-MS/MS, leading to the identification of 2764 metabolites, which were categorized into 17 classes.

Metabolite profiling was performed using an LC-MS/MS system to identify differential metabolites in new and mowed *G. uralensis* plants in response to *B. tabaci* infestation. Across the different treatment groups, a total of 2764 metabolites were identified and classified into 17 categories (Figure 6). The relative abundances of these metabolite classes, in descending order, were as follows: phenylpropanoids and polyketides (22.29%), lipids and lipid-like molecules (19.43%), organic acids and derivatives (14.94%), organooxygen compounds (14.69%), organoheterocyclic compounds (13.49%), and benzenoids (9.30%).

### 3.7. KEGG Analysis of Differential Metabolite

**Figure 7:**
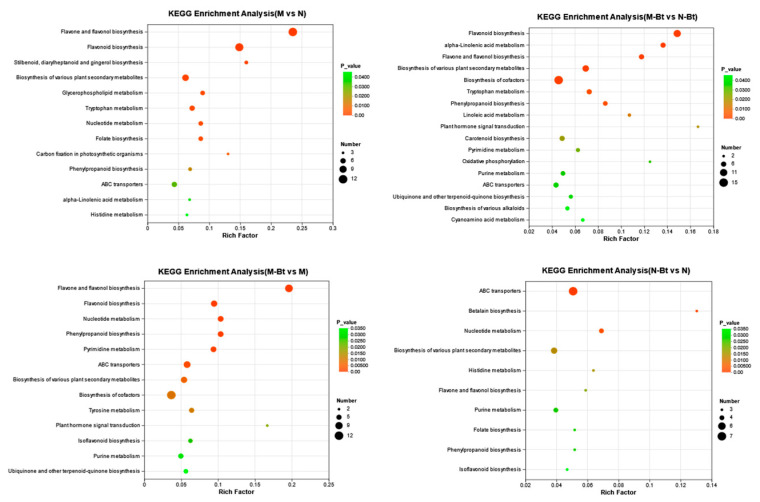
KEGG pathway enrichment analysis bubble plots of differential metabolites (DEMs) in four pairwise comparison groups of *G. uralensis* samples. The four subplots (arranged top-left to bottom-right) correspond to the following pairwise comparisons: M vs. N, M-Bt vs. N-Bt, M-Bt vs. M, and N-Bt vs. N. In each bubble plot: The *x*-axis represents the Rich factor (ratio of DEMs annotated to a KEGG pathway to the total background metabolites annotated to that pathway, reflecting enrichment degree); The *y*-axis lists significantly enriched KEGG pathways (screened with *p* < 0.05); Bubble color corresponds to *p*-value (darker shades indicate a smaller *p*-value, i.e., more significant enrichment); Bubble size represents the number of DEMs annotated to the corresponding KEGG pathway.

In the M vs. N group, a total of 639 DEMs were identified and enriched in 62 metabolic pathways, with 13 pathways showing significant differences (*p* < 0.05), primarily including flavone and flavonol biosynthesis and flavonoid biosynthesis. In the M-Bt vs. N-Bt group, 595 DEMs were identified and enriched in 64 pathways, with 17 pathways showing significant differences (*p* < 0.05), including flavonoid biosynthesis, α-linolenic acid metabolism, and flavone and flavonol biosynthesis. In the M-Bt vs. M group, 619 DEMs were identified and enriched in 60 pathways, with 13 pathways showing significant differences (*p* < 0.05), including flavone and flavonol biosynthesis, flavonoid biosynthesis, and phenylpropanoid biosynthesis. In the N-Bt vs. N group, 417 DEMs were identified and enriched in 54 pathways, with 10 pathways showing significant differences (*p* < 0.05), including ATP-binding cassette (ABC) transporters, betalain biosynthesis, and nucleotide metabolism. Notably, in the M vs. N group, all DEMs enriched in flavone and flavonol biosynthesis and flavonoid biosynthesis were significantly upregulated. The identified DEMs in M-Bt vs. M and N-Bt vs. N groups were significantly enriched in flavone and flavonol biosynthesis, ABC transporters, phenylpropanoid biosynthesis, and isoflavonoid biosynthesis pathways. Among these, flavone and flavonol biosynthesis and ABC transporter pathways exhibited significant enrichment across all treatment groups (Figure 7).

### 3.8. Combined Analysis of Transcriptomic and Metabolomic Data

**Figure 8:**
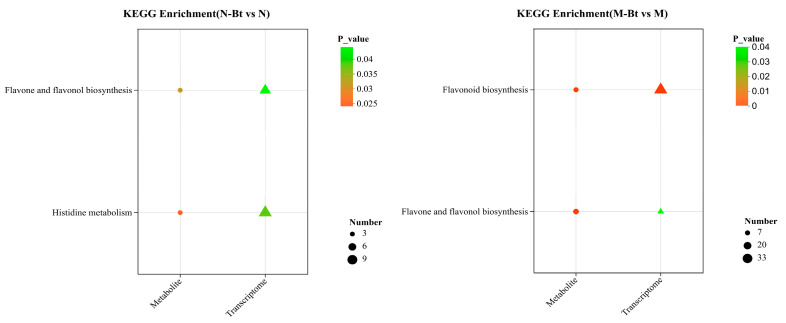
Combined KEGG pathway enrichment analysis bubble plots of DEGs and DEMs in N-Bt vs. N and M-Bt vs. M. In each bubble plot: Circles represent the metabolome, and triangles represent the transcriptome. The color of the bubble corresponds to the *p*-value (a darker color indicates a smaller *p*-value, i.e., more significant enrichment).

To further elucidate the response mechanisms of *G. uralensis* to *B. tabaci* infestation, A combined analysis was conducted based on DEGs and DEMs. In the absence of *B. tabaci* infestation, there were no commonly enriched pathways between DEMs and DEGs in the M vs. N group. However, after *B. tabaci* feeding, the DEMs and DEGs between M-Bt vs. the M group were co-enriched in flavonoid biosynthesis and flavone and flavonol biosynthesis pathways, with major DEMs such as taxifolin, naringenin chalcone, hesperetin, and kaempferol. In the N-Bt vs. N group, the co-enriched pathways were histidine metabolism and flavone and flavonol biosynthesis, with key DEMs including L-histidine, luteolin, rutin, and acacetin. Notably, both M-Bt vs. M and N-Bt vs. N groups showed significant co-enrichment of DEMs and DEGs in the flavone and flavonol biosynthesis pathway (Figure 8).

### 3.9. Defense Pathways of G. uralensis in Response to B. tabaci Feeding

#### 3.9.1. Terpenoid Skeleton Biosynthesis Pathways

**Figure 9:**
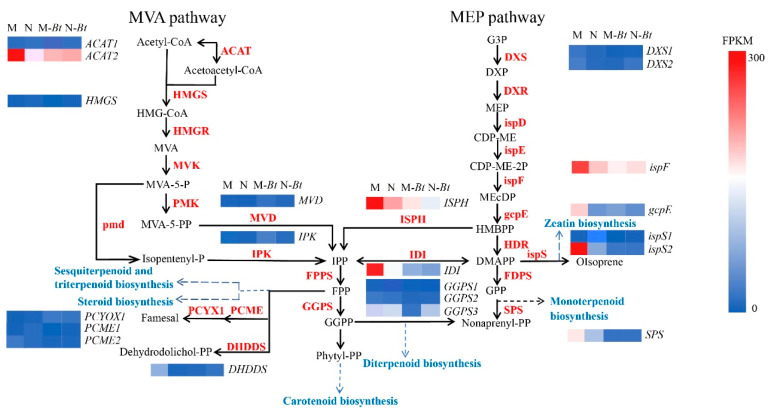
Terpenoid skeleton biosynthesis pathways and expression profiles of related DEGs in *G. uralensis* samples. Key enzymes in the Terpenoid skeleton pathways are labeled with their corresponding genes; arrows indicate metabolic reactions, and dashed arrows link to downstream terpenoid sub-biosynthesis pathways.

Based on the GC-MS analysis results, mowed *G. uralensis plants* contained a high abundance of terpenoid compounds, which may be the primary reason for the significant feeding preference of *B. tabaci* toward new and mowed *G. uralensis*. KEGG enrichment analysis of the Terpenoid skeleton biosynthesis pathways identified 21 significantly enriched DEGs in this pathway (Figure 9). Key genes included *2-C-methyl-D-erythritol 2,4-cyclodiphosphate synthase* (*ispF*), *isopentenyl-diphosphate delta-isomerase* (*idi*), *4-hydroxy-3-methylbut-2-en-1-yl diphosphate reductase* (*ispH*), and *isoprene synthase* (*ispS*), all of which exhibited high expression levels and FPKM values. Gene expression levels were reflected by transcript abundance, with higher transcript abundance indicating higher gene expression.

#### 3.9.2. SA and JA Signaling Pathways

**Figure 10:**
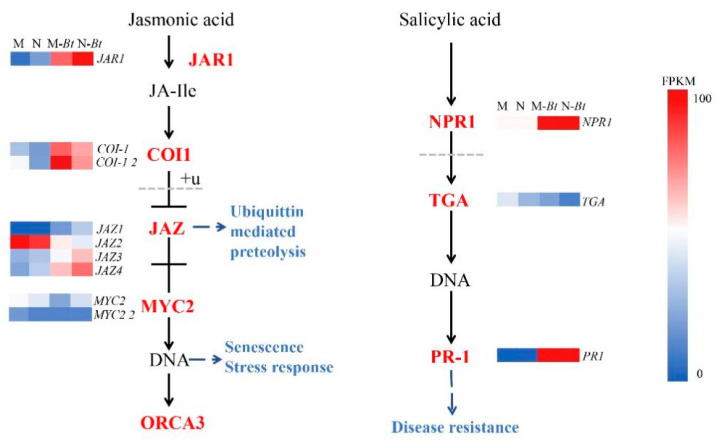
JA and SA signaling pathways with expression profiles of related DEGs in *G. uralensis* samples. This figure displays two plant hormone signaling pathways and the FPKM-based expression profiles of related DEGs.

Through integrated transcriptomic and metabolomic analysis of all groups, we observed that both SA and JA signaling pathways were differentially enriched in M-Bt vs. M and N-Bt vs. N groups. As shown in Figure 9 and Figure 10 DEGs were enriched in the JA pathway, including the *jasmonate-resistant 1* gene (*JAR1*) and *coronatine insensitive 1* gene (*COI1*), which were significantly upregulated. In the SA pathway, 3 DEGs were enriched, with key regulatory genes such as *non-expressor of pathogenesis-related genes 1* (*NPR1*) and *pathogenesis-related protein 1* (*PR1*) showing significant upregulation and high expression abundance.

#### 3.9.3. Isoflavone Biosynthesis Pathway

**Figure 11:**
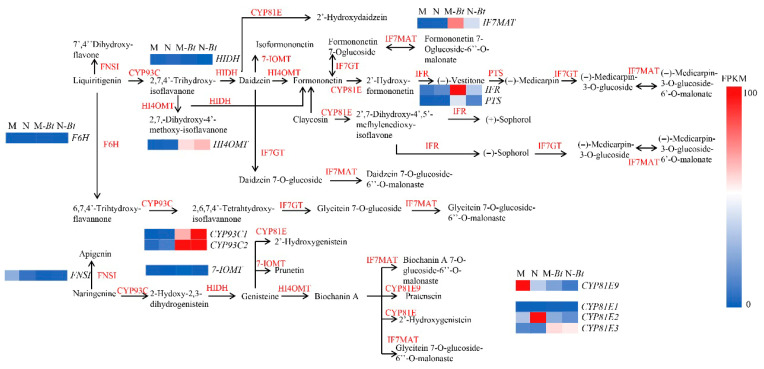
Isoflavonoid biosynthesis pathway and expression profiles of related genes in *G. uralensis* samples. This figure illustrates the branched isoflavonoid biosynthesis pathway and FPKM-based expression profiles of related genes across four groups.

The isoflavonoid biosynthesis pathway represents a complex and highly branched metabolic process involving multiple key metabolites and enzymes. As illustrated in Figure 11, the initial metabolite 7,4′-dihydroxyflavone is converted to liquiritigenin through the action of F6H and FNSI enzymes, which is subsequently transformed by CYP93C into 2,7,4′-trihydroxyisoflavanone. This intermediate is then methylated by HI4OMT to produce daidzein, a pivotal intermediate in isoflavonoid biosynthesis that serves as the substrate for various downstream modifications. Through the catalytic activities of CYP81E, IF7GT, and IF7MAT enzymes, daidzein can be further metabolized into diverse isoflavonoids and their derivatives, including 2′-hydroxydaidzein, formononetin 7-O-glucoside-6′′-O-malonate, and (−)-medicarpin. Following *B. tabaci* infestation, the isoflavonoid biosynthesis pathway exhibited enrichment of 14 genes, with the key regulatory enzyme gene *2′-hydroxyisoflavanone reductase* (*IFR*) showing significant upregulation specifically in mowed plants. Notably, other critical biosynthetic genes, including *HI4OMT*, *CYP93C1*, *CYP93C2*, and *IF7MA*, demonstrated upregulated expression in both new and mowed *G. uralensis* plants under insect stress conditions.

### 3.10. qRT-PCR Validation

**Figure 12:**
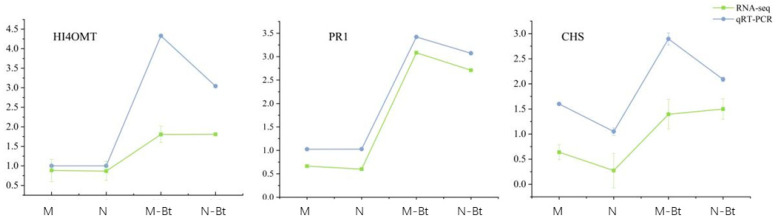
Validation of RNA-seq results by qRT-PCR for three differentially expressed genes in *Glycyrrhiza uralensis* samples. This figure contains three line plots (from (**left**) to (**right**)) showing the expression patterns of genes *HI4OMT*, *PR1*, and *CHS* across four sample groups. The green line represents expression levels from RNA-seq analysis; the blue line represents expression levels quantified by qRT-PCR.

To validate the reliability of the transcriptomic data, we quantified the transcript levels of three significantly differentially expressed genes (*HI4OMT*, *PR1*, and *CHS*) using qRT-PCR. The results demonstrated that the expression patterns of these genes were highly consistent with the RNA-seq analysis (Figure 12). The expression trends of the candidate genes in qRT-PCR closely matched the relative expression levels obtained from Illumina RNA-Seq, indicating high reproducibility and accuracy of the RNA-Seq results. Thus, the RNA-seq analysis not only demonstrated high reproducibility and reliability but also provided valuable insights for subsequent studies on key genes involved in salicylic acid and jasmonic acid signaling pathways, as well as the accumulation mechanisms of flavonoids and isoflavonoids.

## 4. Discussion

Compared with mowed *G. uralensis*, *Bemisia tabaci* exhibited a significant feeding preference for new plants, indicating that mowing treatment effectively enhances resistance against whitefly infestation. This enhanced resistance was closely associated with mowing-induced alterations in volatile organic compound (VOC) profiles. In new *G. uralensis*, alcohols constituted the dominant class of VOCs (40.66%), with cis-3-hexen-1-ol (20.04%) and trans-3-hexen-1-ol (18.10%) being the most abundant components. Numerous studies have demonstrated that cis-3-hexen-1-ol acts as a strong attractant for *B. tabaci* and other piercing–sucking insects, including *Plutella xylostella*, *Aphidius gifuensis*, and *Empoasca flavescens*, thereby playing a key role in host location [24,25,26,27,28].

In contrast, terpenoids dominated the VOC profile of mowed plants (57.10%), particularly 3-carene (18.65%) and β-pinene (21.44%). These compounds have been reported to repel *B. tabaci* and other insect species, such as *Eupithecia abietaria*, *Debrunneata staudinger*, and *Dendroctonus pseudotsugae*, thereby interfering with insect host-selection behavior [29,30]. Notably, the attractive and repellent roles of these VOCs are consistent with our previous olfactory behavioral assays, which demonstrated that cis-3-hexen-1-ol and trans-3-hexen-1-ol attract *B. tabaci*, whereas 3-carene and β-pinene exhibit significant repellent effects [31]. At the molecular level, the high expression of 15 differentially expressed genes (DEGs), including *DXS*, *idi*, *ispF*, and *ispH*, further supports enhanced terpenoid biosynthesis in mowed *G. uralensis*. Among these, *DXS*, *ispF*, and *ispH* are key rate-limiting enzymes in the methylerythritol phosphate (MEP) pathway, which generates the universal terpenoid precursors isopentenyl diphosphate (IPP) and dimethylallyl diphosphate (DMAPP) [21]. Upregulation of these genes likely promotes the synthesis and emission of terpenoids, contributing to enhanced repellence against *B. tabaci* [32].

Beyond volatile-mediated resistance, mowing also induced substantial changes in primary metabolism and cellular activity. Transcriptomic analysis revealed that DEGs in the mowing versus non-mowing comparison (M vs. N) were predominantly enriched in ribosome, ribosome biogenesis in eukaryotes, starch and sucrose metabolism, pyrimidine metabolism, and riboflavin metabolism pathways. These pathways are mainly associated with protein synthesis, nucleotide metabolism, and energy production, suggesting that mowing-induced mechanical damage primarily activates basal metabolic reprogramming and wound-recovery processes rather than specialized insect defense pathways.

In contrast, transcriptomic responses induced by *B. tabaci* infestation differed markedly from those triggered by mechanical damage alone. DEGs identified in both the mowed plants under whitefly infestation (M-Bt vs. M) and the new plants under infestation (N-Bt vs. N) were significantly enriched in flavonoid biosynthesis, flavone and flavonol biosynthesis, plant circadian rhythm regulation, starch and sucrose metabolism, and MAPK signaling pathways. This convergence indicates that whitefly feeding activates conserved insect-responsive defense programs irrespective of mowing treatment. However, distinct differences were also observed between the two infestation conditions. In the M-Bt vs. M group, DEGs were additionally enriched in ribosome and ribosome biogenesis pathways, whereas in the N-Bt vs. N group, DEGs were significantly enriched in valine, leucine, and isoleucine degradation pathways. These differences suggest that mowing primes plants for a more rapid and efficient transcriptional response upon insect attack, whereas non-mowed plants rely more heavily on metabolic reallocation to cope with herbivory stress.

Taken together, these contrasting enrichment patterns provide strong evidence that mechanical damage caused by mowing and stylet-mediated feeding by *B. tabaci* activate fundamentally different regulatory programs. Mechanical damage mainly affects growth-related and primary metabolic pathways, whereas whitefly feeding—through the combined effects of stylet penetration and salivary elicitors—induces specialized defense pathways. When insect-derived elicitors are considered, pronounced differences emerge in chemical profiles, gene expression patterns, and phytohormone signaling, highlighting the necessity of distinguishing herbivore-specific elicitation from mechanical injury when interpreting plant defense responses.

Consistent with these transcriptomic patterns, flavonoid metabolism emerged as a central component of *G. uralensis* defense against *B. tabaci*. Transcriptomic and metabolomic analyses revealed significant upregulation of key biosynthetic genes, including *CHS* and *HCT*, along with increased accumulation of flavonoid metabolites such as quercetin, kaempferol, syringetin, and isoquercitrin. Flavonoids and flavonols are well known to function as direct toxins, feeding deterrents, and antioxidants that mitigate herbivore-induced oxidative stress [33,34]. Previous studies have similarly demonstrated that flavonols contribute to resistance against *Anticarsia gemmatalis* in soybean [35], while rutin and quercetin-3-glucoside inhibit larval development in pine caterpillars [36]. In addition, flavonoids can suppress insect oviposition, as evidenced by the inhibitory effects of quercetin-3-O-rutinoside on *Pieris rapae* [37].

Following *B. tabaci* infestation, ester volatiles such as (3Z)-hex-3-en-1-yl acetate and cis-3-hexenyl 2-methylbutanoate increased significantly in both new and mowed plants, with a more pronounced induction in mowed plants. These compounds have been reported to act as attractants for *B. tabaci* and other insects [38,39]. Unlike mowing-induced terpenoid shifts, the induction of these ester volatiles is more likely associated with whitefly-derived salivary elicitors rather than mechanical damage alone, further supporting the existence of insect-specific biosynthetic responses.

At the signaling level, enrichment of the MAPK signaling pathway in both infestation comparisons highlights its central role in transducing whitefly-derived signals. MAPK cascades integrate early perception events with phytohormone signaling, particularly salicylic acid (SA) and jasmonic acid (JA) pathways [40]. Although SA and JA signaling pathways are often considered antagonistic, synergistic and additive interactions have been widely reported, particularly in response to insect herbivory [41,42]. In this study, key SA signaling genes (*NPR1* and *PR-1*) and JA signaling genes (*JAR1* and *COI1*) were significantly upregulated following *B. tabaci* infestation, indicating coordinated activation of both pathways [43,44]. Notably, WRKY transcription factors have been reported to modulate plant immunity against whitefly infestation by interacting with MAPK cascade pathways, thereby linking MAPK signaling with transcriptional reprogramming during defense responses [45].

Metabolomic analyses further revealed that *B. tabaci* infestation significantly enriched differentially expressed metabolites in flavone and flavonol biosynthesis, isoflavonoid biosynthesis, ABC transporter pathways, and phenylpropanoid metabolism. Isoflavonoids, which are characteristic of leguminous plants, function as phytoalexins and play important roles in resistance to biotic and abiotic stresses [46,47,48]. The coordinated upregulation of genes such as *IFR*, *CYP93C1*, *CYP93C2*, *HI4OMT*, and *IF7MA* suggests comprehensive activation of the isoflavonoid pathway from precursor synthesis to end-product modification, consistent with previous reports in soybean and tea plants under insect attack [49,50].

Finally, integrated transcriptomic and metabolomic analyses demonstrated that flavonol biosynthesis represents a core metabolic pathway underlying *G. uralensis* resistance to *B. tabaci*. Although overlap between transcriptomic and metabolomic KEGG enrichment was limited, this likely reflects temporal delays between transcriptional regulation and metabolite accumulation, as well as the multilayered nature of plant defense responses [51]. Collectively, these findings demonstrate that mowing primes *G. uralensis* for enhanced defense, while whitefly-derived elicitors activate distinct molecular, metabolic, and hormonal responses that together shape effective resistance against herbivory.

## 5. Conclusions

In conclusion, mowing treatment markedly enhances the resistance of *Glycyrrhiza uralensis* against *Bemisia tabaci* through coordinated regulation of volatile emissions, secondary metabolite biosynthesis, and defense signaling pathways. Mowing fundamentally reshapes the VOC profile of *G. uralensis*, shifting it from green leaf volatiles that attract whiteflies to terpenoid-dominated blends with strong repellent effects, a process supported by the upregulation of key genes in the MEP pathway. In parallel, mowing primes plants for enhanced chemical defense, promoting the accumulation of flavonoids and isoflavonoids that function as toxins, feeding deterrents, and antioxidants during whitefly infestation.

Moreover, *B. tabaci* feeding induces a systemic defense response in *G. uralensis* that is distinct from mechanical damage alone, characterized by activation of MAPK signaling and coordinated salicylic acid and jasmonic acid pathways. Integrated transcriptomic and metabolomic analyses identified flavonol biosynthesis as a core metabolic pathway underlying licorice resistance to whitefly attack.

Overall, this study demonstrates that mowing acts as an effective agronomic practice to prime multi-layered defense responses in *G. uralensis*, thereby enhancing resistance to *B. tabaci*. These findings deepen our understanding of plant–insect interactions and provide a theoretical basis for developing sustainable pest management strategies based on crop management practices such as timely mowing.

## Figures and Tables

**Table 1:** Developmental duration and adult longevity of *Bemisia tabaci* on new and mowed *Glycyrrhiza uralensis*.

Treatment	Egg (d)	1st Instar (d)	2nd Instar (d)	3rd Instar (d)	4th Instar (d)	Egg–Adult (d)	Adult Longevity (d)
New plants	7.00 ± 0.90	3.05 ± 0.61	2.02 ± 0.02	4.05 ± 0.06	5.98 ± 0.06	22.11 ± 0.13	31.33 ± 1.52
Mowed plants	8.01 ± 0.11	3.02 ± 0.15	2.04 ± 0.04	3.98 ± 0.06	6.06 ± 0.11	23.22 ± 0.25	31.50 ± 0.50

Note: Values are presented as mean ± SD (*n* = 3). Developmental duration includes egg stage, nymphal instars, and total egg-to-adult period. Adult longevity was assessed for F1 adults reared on new or mowed plants under controlled conditions.

**Table 2:** Volatile components and relative contents in *G. uralensis* leaves under different treatments.

Types	Name	N	M	N-Bt	M-Bt	*p*	VIP
Aldehydes	Heptaldehyde	0.31 ± 0.50 b	0.35 ± 0.83 b	0.45 ± 0.22 ab	0.73 ± 0.35 a	0.015	0.238
	Octanal	0.36 ± 0.04 a	0.18 ± 0.04 b	0.42 ± 0.13 a	0.30 ± 0.10 ab	0.019	0.170
	Hexanal	0.26 ± 0.91 b	0.22 ± 0.12 b	0.21 ± 0.19 b	1.43 ± 0.45 a	0.001	0.522
	Isovaleraldehyde	0.59 ± 0.06 b	0.91 ± 0.39 b	0.82 ± 0.34 b	1.85 ± 0.21 a	0.003	0.444
	Valeraldehyde	0.11 ± 0.01 c	0.65 ± 0.13 b	1.01 ± 0.19 a	0.53 ± 0.12 b	0.000	0.593
	Benzaldehyde	NA	0.18 ± 0.01 b	NA	0.22 ± 0.01 a	0.000	0.193
	Nonanal	0.29 ± 0.14	2.64 ± 0.83	1.29 ± 1.66	1.48 ± 1.20	0.160	0.759
	Decanal	0.17 ± 0.04 b	NA	1.44 ± 0.49 a	NA	0.000	0.692
Alcohols	Pentanol	1.13 ± 0.50 b	1.42 ± 0.11 b	1.46 ± 0.17 b	3.01 ± 1.21 a	0.022	0.523
	trans-1,2-Cyclopentanediol	0.58 ± 0.10 b	1.55 ± 0.76 a	0.23 ± 0.50 b	0.79 ± 0.59 ab	0.017	0.389
	trans-3-Hexen-1-ol	20.04 ± 0.43 a	2.18 ± 0.24 b	22.18 ± 4.38 a	2.45 ± 0.46 b	0.000	1.859
	cis-3-Hexen-1-ol	18.91 ± 0.34 b	1.12 ± 0.21 c	21.79 ± 1.70 a	1.11 ± 0.18 c	0.000	1.946
Terpenes	3-Carene	8.83 ± 1.01 b	18.65 ± 1.26 a	7.79 ± 0.89 b	2.25 ± 0.72 c	0.000	1.874
	α-Pinene	NA	2.66 ± 0.26 ab	0.73 ± 0.44 b	4.60 ± 3.10 a	0.028	0.842
	(+)-α-Pinene	0.26 ± 0.16 b	4.92 ± 3.88 a	NA	4.07 ± 0.73 ab	0.031	0.850
	β-Pinene	5.17 ± 1.25 bc	21.44 ± 0.90 a	1.31 ± 0.15 c	9.34 ± 5.60 b	0.000	1.708
	p-Mentha-1,4-diene	0.23 ± 0.11 b	1.43 ± 0.21 a	NA	NA	0.000	0.520
	(+)-Limonene	1.29 ± 0.15 c	1.81 ± 0.62 bc	2.31 ± 0.22 ab	2.76 ± 0.09 a	0.004	0.505
	α-Terpinene	0.11 ± 0.01 a	NA	NA	NA	0.000	0.205
	α-Copaene	0.27 ± 0.06 d	1.36 ± 0.10 b	0.87 ± 0.07 c	2.05 ± 0.65 a	0.000	0.512
	trans-Caryophyllene	0.18 ± 0.10 d	1.34 ± 0.20 b	0.84 ± 0.13 c	1.97 ± 0.23 a	0.000	0.521
	Alloaromadendrene	NA	1.30 ± 0.16	NA	NA	0.000	0.538
	Valencene	0.27 ± 0.05 b	2.28 ± 0.21 a	0.31 ± 0.02 b	2.42 ± 0.09 a	0.000	0.611
Esters	(3Z)-Hex-3-en-1-yl acetate	5.34 ± 1.31 b	5.31 ± 1.14 b	27.76 ± 9.50 a	22.37 ± 9.99 ab	0.008	2.474
	Linalyl acetate	0.37 ± 0.10	3.40 ± 4.92	NA	NA	0.327	0.724
	cis-3-Hexenyl isobutyrate	0.86 ± 0.48 c	1.85 ± 0.22 ab	1.54 ± 0.58 bc	2.58 ± 0.36 a	0.007	0.458
	Cis-3-Hexenyl Butyrate	0.84 ± 0.17 b	3.06 ± 0.02 a	1.44 ± 0.79 ab	3.10 ± 1.09 a	0.007	0.594
	Cis-3-Hexenyl 2-Methylbutanoate	0.77 ± 0.20 c	4.91 ± 1.39 bc	8.79 ± 0.76 b	24.95 ± 5.54 a	0.000	1.984
Acids	Oxalic acid	0.43 ± 0.25 b	1.46 ± 0.02 a	NA	NA	0.000	0.508
	Malonic acid	0.14 ± 0.26 b	0.83 ± 0.27 a	0.16 ± 0.05 b	0.37 ± 0.17 b	0.003	0.323
Others	(−)-Isoborneolaceticacid	NA	NA	0.57 ± 0.77	NA	0.251	0.420

Note: N = new *G. uralensis* plants; M = mowed *G. uralensis* plants; N-Bt = new *G. uralensis* plants infested by *B. tabaci*; M-Bt = mowed *G. uralensis* plants infested by *B. tabaci*. Values in the table represent the relative content of volatile compounds (unit: %) calculated by the peak area normalization method, and data are expressed as mean ± standard deviation. Different lowercase letters (a–d) in the same row indicate significant differences among groups (*p* < 0.05). NA indicates that the compound was not detected in the corresponding group. *p*-value represents the significance of differences in compound content among groups; VIP (Variable Importance in Projection) value is derived from orthogonal partial least squares discriminant analysis (OPLS-DA). Differential volatile compounds were screened with the criteria of *p* < 0.05 and VIP > 1.

## Data Availability

The raw sequence data reported in this paper have been deposited in the Genome Sequence Archive (Genomics, Proteomics & Bioinformatics 2025) in National Genomics Data Center (Nucleic Acids Res 2025), China National Center for Bioinformation/Beijing Institute of Genomics, Chinese Academy of Sciences (GSA: CRA034713) that are publicly accessible at https://ngdc.cncb.ac.cn/gsa (accessed on 5 December 2025). The metabolomic data reported in this paper have been deposited in the OMIX, China National Center for Bioinformation/Beijing Institute of Genomics, Chinese Academy of Sciences (https://ngdc.cncb.ac.cn/omix: accession no. OMIX013501, accessed on 5 December 2025). All other supporting data are included in the article or have been submitted as Supplementary Materials.

## Supplementary Materials

The following supporting information can be downloaded at: https://www.mdpi.com/article/10.3390/insects17020211/s1. Figure S1: Evaluate the dispersion degree of transcriptomes across all samples via Principal Component Analysis (PCA); Table S1. Primer sequences; Table S2. Quality control of transcriptome data.
