# Supplementary material for: Mowing Enhances Insect Resistance in Glycyrrhiza uralensis by Reprogramming Volatile Profiles and Inducing Flavonoid Accumulation
Source: Insects. 2026 Feb 17;17(2):211. doi: 10.3390/insects17020211 (PMC12940751; doi:10.3390/insects17020211)
Supplement: Supplementary file 1 [file insects-17-00211-s001.zip › TableS1.docx]

| TableA1. Primer sequences | | |
| --- | --- | --- |
| Primer name | Primer sequence | Amplification  product length（bp） |
| JAR1-F | CACCAGTTCTCGCAAAGTC | 110 |
| JAR1-R | AACAGCGGTTCCTAATCC |  |
| HI4OMT-F | CCAAAGTCGGTGTCCTCTA | 247 |
| HI4OMT-R | TTGTCCTCGTTGAACCATT |  |
| PR1-F | CTACCTGGACGCCCACAAC | 184 |
| PR1-R | TGTTCCCGTGAGGTCAGCA |  |
| CHS-F | AAGTCCAAGATTACCCACC | 215 |
| CHS-R | TCTGAACAAACCACGAGCA |  |
