# Supplementary material for: Mowing Enhances Insect Resistance in Glycyrrhiza uralensis by Reprogramming Volatile Profiles and Inducing Flavonoid Accumulation
Source: Insects. 2026 Feb 17;17(2):211. doi: 10.3390/insects17020211 (PMC12940751; doi:10.3390/insects17020211)
Supplement: Supplementary file 1 [file insects-17-00211-s001.zip › TableS2.docx]

| **TableA2**. Quality control of transcriptome data | | | | | | | |
| --- | --- | --- | --- | --- | --- | --- | --- |
| **Sample name** | **Number of effective reads** | **Number of bases after filtering** | **Q20(%)** | **Q30 (%)** | **GC (%)** | **Total number of matches** | **Match rate (%)** |
| M-1 | 53847690 | 8037822864 | 98.66 | 95.72 | 44.53 | 50165214 | 93.16 |
| M-2 | 51391132 | 7702499048 | 98.63 | 95.59 | 44.59 | 47934691 | 93.27 |
| M-3 | 61541224 | 9211208241 | 98.71 | 95.81 | 44.27 | 57050837 | 92.7 |
| N-1 | 40571454 | 6078233095 | 98.73 | 95.86 | 44.15 | 37905542 | 93.43 |
| N-2 | 49527494 | 7398082416 | 98.72 | 95.86 | 44.18 | 46171850 | 93.22 |
| N-3 | 47680160 | 7097718842 | 98.61 | 95.57 | 43.98 | 44412034 | 93.15 |
| M-B-1 | 57676166 | 8552130357 | 98.72 | 95.84 | 44.36 | 54018935 | 93.66 |
| M-B-2 | 51092882 | 7618506933 | 98.69 | 95.73 | 44.02 | 47955304 | 93.86 |
| M-B-3 | 46885062 | 6982718899 | 98.67 | 95.67 | 43.58 | 43775296 | 93.37 |
| N-B-1 | 48847284 | 7214632004 | 98.73 | 95.87 | 43.72 | 45314098 | 92.77 |
| N-B-2 | 54261312 | 8007933969 | 98.62 | 95.64 | 43.84 | 50357963 | 92.81 |
| N-B-3 | 45885432 | 6821234970 | 98.62 | 95.56 | 43.52 | 42481903 | 92.58 |
