# Supplementary figures and images for: Mowing Enhances Insect Resistance in Glycyrrhiza uralensis by Reprogramming Volatile Profiles and Inducing Flavonoid Accumulation
Source: Insects. 2026 Feb 17;17(2):211. doi: 10.3390/insects17020211 (PMC12940751; doi:10.3390/insects17020211)

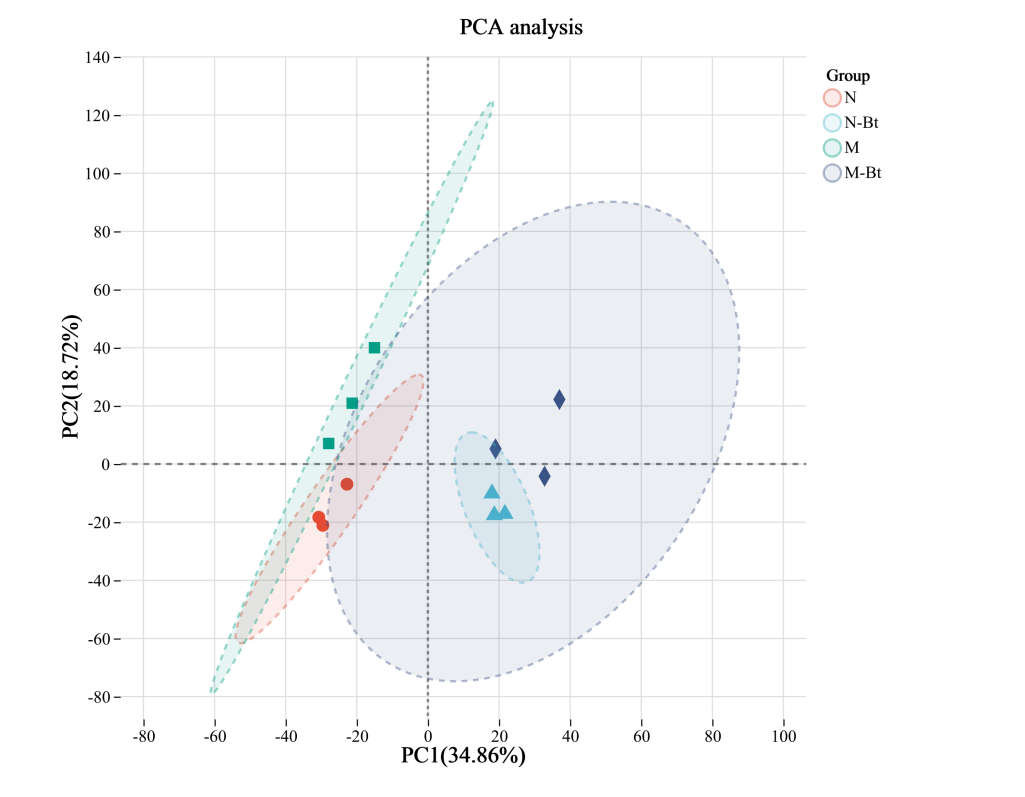

Supplement: Supplementary file 1 [file insects-17-00211-s001.zip › FigureS1.tif]
